# ‘You Also Have to Let People Go’—Effects of Formative Experiences with Dying and Death on Medical Trainees’ Attitudes

**DOI:** 10.1007/s40670-024-02090-0

**Published:** 2024-06-12

**Authors:** Nana Jedlicska, Carolin Rossmanith, Sabrina Lichtenberg, Dagmara Srnová, Marjo Wijnen-Meijer, Martin Gartmeier, Pascal O. Berberat

**Affiliations:** 1 TUM Medical Education Center, TUM School of Medicine and Health, Technical University of Munich, Ismaninger Straße 22, 81675 Munich, Germany; 2 Institute of Medical Education, Medical Faculty and University Hospital Carl Gustav Carus, TUD Dresden University of Technology, Fetscherstraße 74, 01307 Dresden, Germany

**Keywords:** Dying and death, Medical students, Medical residents, End-of-life care, Role of physician

## Abstract

This study investigates the effects of medical students’ and residents’ formative patient death experiences on their understanding of the role of the physician in dealing with dying patients. Analyses revealed a change in attitude, an acceptance of death as ‘part of life’. Thoughtful and comprehensive care, allowing patients to die and enabling them to have a beautiful death, were identified as the physician’s duty. Honesty, well timing and completeness were determined as the guiding principles of communication. The importance of distancing and keeping in control to practice the medical profession was stressed. The burdensome nature of making therapeutic decisions was emphasized.

## Introduction

Numerous studies illustrate the demanding nature of encounters with dying and death for physicians [1–4]. These studies analyse physicians’ dealing with death from different perspectives. Existing research has elicited physicians’ formative experiences regarding dying and death. First encounters with patient death [3, 5, 6], good death [7], unexpected death [6–8] and overtreated death [7] are described as particularly challenging. The circumstances of death such as the age of the patient [5, 8, 9], personal attachment to the dying person [2, 6, 7, 10] and perception of one’s own role in the dying event [1, 9] are discussed as factors that determine the formative character of these experiences.

Various studies explore physicians’ emotional, cognitive and physical reactions to the patients’ death [1, 5]. The emotions described encompass shock [3, 5, 7, 11], distress [7], helplessness [5, 8, 12], anger [5, 8, 13], guilt [1, 4, 7, 8, 13, 14], sadness [1, 3–5, 8, 11–13, 15] grief [2, 4, 5], uncertainty [8], satisfaction [3] and feeling uplifted [11]. On the cognitive level, the death of the patient leads physicians to question the care provided [8, 13], their competence [7] and think about the dying experience and the idea of death [5]. The physical reactions to dying experiences include, among others, sleep disorders [5]. The research also shows that medical students feel ill-prepared by their medical training to encounter the death of a patient [6, 8, 16–18] and insufficiently supported by their supervisors concerning end-of-life (EOL) issues [8, 19]. The lack of discussion culture in the hospital environment on the subject of dying and death is criticized [15].

Much of the research focuses on coping mechanisms. Common coping strategies described in the literature include talking to someone [1, 3, 5–7, 10, 20], crying [1], carrying on with work [1, 3, 6], engaging in distracting activities [2, 3, 5, 20], rationalizing [1, 11], normalizing [6], self-reflection, self-care, finding work-life balance [8] and turning to religion [1, 3, 4, 6, 10]. There is evidence in the literature that medical students commonly experience a lack of reflection [3, 7], which is attributed to systemic factors such as time pressure and heavy workload [10]. At the same time, the research emphasizes the importance of peer and senior support, including debriefing opportunities [8, 11]. The importance of family and external networks is also emphasized [8].

Furthermore, existing studies show that experiences with dying and death affect physicians’ personal and professional development [10, 20, 21, 22, 23]. Witnessing the moment of death represents a ‘turning point’ in the professional lives of aspiring physicians [15] and is critical in shaping physicians’ future care of patients [7]. This development includes becoming desensitized to the human dimension of care [20, 22]. In this context, the tension between emotional involvement and professional detachment is emphasized [20, 24]. Exposure to death leads physicians to gain deeper knowledge [25] and greater confidence in their own EOL skills [10, 12, 26] and an understanding of their professional role and responsibilities in processing the formalities of death [20]. Research reports changes in medical students’ and residents’ perception of the role of the physician from the curative perspective to the caring view [8, 20]. Various factors such as positive role modelling and spirituality can facilitate this transformation [8]. Kuczewski et al. provide insight into the skills and attitudes medical students found most relevant in meeting the spiritual needs of dying patients and their relatives and emphasize the importance of clear communication, comprehensive care and tailoring treatment plans to meet the patient’s therapy goals [22]. The importance of empathy and seeing the patient as a ‘whole’ person and not ‘a set of symptoms’ is outlined [24]. The changes in the view of death as a part of life and the changes in view of the role of medicine from trying to cheat death to preserving the quality of life are reported [20, 27].

Despite the richness of the existing literature, we argue that there is a lack of research that examines the effects of encounters with dying and death on physicians’ perception of their own role in dealing with dying patients. Considering the relevance of the topic, we sought to extend previous research by investigating the effects of the formative patient death experiences on medical students’ and residents’ attitudes, focusing on the following research question: How do medical students and residents understand their role as (future) physicians in dealing with dying patients and death as a result of their most formative patient death experiences?

Our study forms part of a larger study that examined medical trainees’ most formative patient death experiences, focusing on characteristics that defined the qualitative character of these experiences [9]. While the large study focused primarily on the perception and experience of the confrontation with death, the current study concentrates on the (long-term) effects of these encounters on (future) physicians’ attitudes.

## Materials and Methods

### Study Sample

The study sample consisted of nine medical students in the second and third trimester in their final year at TUM School of Medicine and nine residents in the midterm of their residency at the TUM University Hospital Rechts der Isar. Due to the increasing clinical exposure and growing responsibility, we considered the final year of the medical school and the residency as particularly important and rich in experience stages on the way to becoming a physician. Initially, all students in the final year of undergraduate medical education were invited to participate in the research through an e-mail advertisement sent to the medical student listserv. Nine medical students expressed their wish to participate and were included in the study. In order to maintain balance, we have decided, based on the number of student participants, to limit the number of residents to nine interviewees. All residents were identified via the respective institute’s website, contacted personally and recruited for the study. We intentionally placed the focus on a heterogeneous group of medical professionals in order to investigate possible differences between students’ and residents’ experiences and thereby enable a comparison between the attitudes of trainees at different stages of medical education. Moreover, we assumed that the character of the clinical experiences would vary depending on the areas of physicians’ expertise. Therefore, we recruited residents from internal medicine (3), surgery (3), paediatrics (2) and anaesthesia and intensive care (1). The participants ranged in age from 24 to 29. Twelve participants were female and six were male. A book voucher was offered as an incentive. The interviewees’ demographics are shown in Table 1.

**Table 1:** Demographics of participants

	**Students** **(*n* = 9)**	**Residents** **(*n* = 9)**	**Total sample** **(*n* = 18)**
**Age in years, mean (range)**			
	25 (24–29)	31 (26–34)	28 (24–34)
**Gender (%)**			
Male	2 (22)	4 (44)	6 (33)
Female	7 (78)	5 (56)	12 (67)
**Area of expertise**			
Internal medicine	–	3	–
Surgery	–	3	–
Paediatrics	–	2	–
Anaesthesia and intensive care	–	1	–

### Data Collection

From December 2013 to April 2014, 18 individual semi-structured interviews were conducted using an interview guideline. At the beginning of each interview, participants were asked to describe their most formative experiences regarding dying and death. In order not to influence the participants’ storytelling, we purposely did not specify a context for these experiences. During the interviews, the interviewees were encouraged to describe the effects of these experiences on their perception of their role as a (future) physician. We used the interview guideline (see Appendix) to obtain responses on relevant topics that were not raised by the interviewees. After the interviews, participants were asked to complete a short sociodemographic questionnaire. Informed consent for involvement in the study was obtained from all participants. Each interview lasted approximately one hour (an average of 57 min, ranging from 45 to 70 min) and was audio taped and transcribed verbatim. Ethical approval was granted for this study from the Ethics Committee of the Faculty of Medicine at Technical University of Munich (Project Number 42/14).

### Analysis

The Qualitative Content Analysis (QCA) approach by M. Schreier (2012) was used to analyse the data [28]. The data analysis consisted of the following steps: we created an initial coding frame as a way of structuring the material and as a filter to scan the material. It consisted of main categories specifying relevant aspects of the material and subcategories specifying the meaning of the material concerning these aspects. The main categories were created deductively, in a concept-driven way by using the interview questions and added subcategories inductively, in a data-driven way based on the material. To generate data-driven categories, two researchers independently open coded four randomly selected interviews—two each with medical students and residents. Adopted from Grounded Theory, this strategy consists of three steps: conceptualizing, defining and developing categories. (1) In the first step of conceptualizing, the researchers closely examined the data, trying to view the data from different perspectives and identify relevant concepts. (2) In the second step of defining categories, similar concepts were grouped together into categories. (3) In the final step, the data categories were arranged as a hierarchical structure and a coding frame was created. In this context, categories were defined, i.e. the rules for assigning data segments to categories were defined. Once all categories had been generated and defined, we revised the coding frame, checking it for any loose ends. The initial coding frame was tried out on six interviews. This iterative process of creating the coding frame was accomplished through frequent group meetings with all stakeholders in which the researchers compared coding results, critically discussed differences and modified categories. Two researchers then applied the coding frame to all the transcripts using MAXQDA 20. Finally, we cross-case analysed each category and wrote memos for each category in order to structure and reflect on the data.

## Results

### The Understanding of the Role of the Physician

Eight themes were identified regarding the impact of the encounters with death on the understanding of the role of the physician in dealing with dying patients and death: *thoughtful care*, *distancing*, *keeping in control*, *communication*, *caring for relatives*, *allowing death*, *enabling a beautiful death* and *making decisions and taking responsibility*. Table 2 provides an overview of the distribution of themes among medical students and residents.

**Table 2:** Distribution of themes among medical students and residents

Theme	Medical students (%)	Residents (%)	Both (%, proportion of all 18 participants)
Thoughtful care	7 (78)	2 (22)	9 (50)
Distancing	4 (40)	6 (60)	10 (56)
Keeping in control	2 (40)	3 (60)	5 (28)
Communication	4 (31)	9 (69)	13 (72)
Caring for relatives	7 (78)	2 (22)	9 (50)
Allowing death	7 (54)	6 (46)	13 (72)
Enabling a beautiful death	7 (54)	6 (46)	13 (72)
Making decisions and taking responsibility	1 (20)	4 (80)	5 (28)

### Thoughtful Care

Seven medical students and two residents considered thoughtful care an essential medical task. Our interviewees agreed that physicians’ duties include not only the treatment of somatic complaints, but also the care of patients’ and their relatives’ emotions and cognitions.

We are not just responsible for giving injections and medications, we are also responsible for what happens to the people. And I think that’s clearly part of our job later as a doctor that you also deal with these things in detail. (D.W., student)

As essential elements of thoughtful care, interviewees stressed empathy, taking time, interacting and respect: Regarding empathy, it was considered critical to recognize the mental and emotional state of patients and their relatives and to empathize with their feelings. Interviewees agreed that physicians should view and respect each patient as an individual with their unique life story and not reduce patients to their disease. The particular importance of respect was furthermore stressed towards patients who are unconscious as well as towards deceased patients’ bodies. Moreover, ‘to be there’ for patients and their relatives and to be a contact person for them for questions was considered important. The lack of time and financial resources was stressed as challenging factors that hinder physicians in providing thoughtful care.

### Distancing

Four students and six residents addressed cognitive and emotional components of distancing as a prerequisite for practicing the medical profession. Cognitive distancing encompasses mental dissociation from the dying experience. Emotional distancing involves the avoidance of emotional attachment to the patients and their relatives and aims at an emotional dissociation from the dying experience. The interviewees explained how encounters with dying people and death caused them to feel ‘sad’ and ‘unhappy’. The participants described long-lasting mental preoccupation and the difficulty of leading a ‘normal life’. Hence, our interviewees emphasized the great importance of distancing for the purpose of ‘self-protection’ in the sense of ‘mental hygiene’. Furthermore, our interviewees highlighted the importance of distancing to make sober therapeutic decisions and not be guided by feelings in the decision-making process.

Aspects which, according to interviewees, make distancing more difficult encompass a sympathetic character and young age of patients and their relatives as well as emotional attachment to them and one’s own personality. Other factors are related to the perception of one’s own role and the interpretation of death as a therapeutic or even personal failure. Moreover, the insecurity concerning therapeutic decisions and actions and the associated doubts make it difficult to maintain distancing. Overall, the interviewees agreed that maintaining emotional and cognitive distance is a matter of experience.

Despite the unified demand for distancing, the interviewees expressed their intention not to lose touch with patients. Several interviewees, especially the medical students, expressed their concern that they would not be able to remain empathic. Various student participants expressed their uncertainty about the relation between emotional involvement and professional detachment.

And I don’t know whether it’s good if you still have emotions involved or whether it wouldn’t really be better if you could look at it all soberly. That’s what I meant at the beginning, I was actually confused as to whether I want to become like that myself, or whether I still want to retain a bit of emotion, because I simply don’t know what is healthier for oneself. (D.W., student)

Overall, our participants emphasized the importance of dealing with the question of balance and one’s personal limits of emotional involvement.

### Keeping in Control

Controlling one’s own emotions and their expression was considered a requirement for physicians by two medical students and three residents. Our interviewees found it perfectly legitimate for physicians to feel emotions themselves; however, the interviewees felt that physicians should keep their compassion under control and that feelings should only be expressed to a moderate extent.

As a physician, you can’t really show too much compassion, that’s just not the role we have. (A.S., resident)

The interviewees stressed the importance of keeping in control in order to ‘stay functional’. The loss of control over one’s own emotions was considered a risk to role reversal in which relatives would then have to console physicians. The interviewees agreed that physicians should remain calm and show ‘strength’.

Our interviewees addressed characteristics that make it difficult to maintain control over one’s own emotions. The circumstances of death such as sudden and young death was described as an aggravating factor. Moreover, similar to the distancing, the patient-related characteristics such as the personality of the patients as well as a close personal relationship to the patients and their relatives were stated as factors that make it more difficult to keep in control. Saying goodbye to the dying patient who is going home to die has been identified as a situation in which maintaining control is particularly difficult as well.

Although interviewees stressed the necessity of maintaining control of one’s own emotions as a physician, they showed great understanding of emotional outbursts, describing them as ‘natural reactions’.

### Communication

Four medical students and nine residents addressed the subject of communication with patients and their relatives as a central medical task. Honesty was stated as the major principle of communication. The residents in particular emphasized how important it is to communicate the patient’s state of health and prognosis honestly in order to involve patients and their relatives in the decision-making process and respect patients’ wishes regarding their own dying. Moreover, according to our interviewees, it is an emotional ‘relief’ to know what the patient’s wishes are and that patients and their relatives have been sincerely informed about the situation.

Alongside communicating honestly, our interviewees, particularly residents, added that well-timed and complete communication of the gravity of the situation was regarded as crucial. Communication must be timely so that those concerned get the opportunity to understand their current situation and to gain an accepting attitude towards the death, to solve unresolved matters and to say goodbye to their loved ones.

Moreover, our interviewees commented on the delivering of bad news. The interviewees emphasized the impact of the way in which bad news is delivered on the perception and experience of patients and their relatives, and deemed the communication of bad news as ‘extremely important’:

I’ve somehow noticed that, as I said, the ‘how’ is often more important than the ‘what’, because the parents or the patients often already know that the news is bad, it’s just how it’s conveyed that’s then often somehow much more important than what’s conveyed to them. (N.H., resident)

The central principles described were to proceed tactfully, with carefully chosen words in order not to deprive patients and relatives of their courage. Nonetheless, it was considered important to address death explicitly, to speak unambiguously and express oneself in sentences that leave no room for doubt and interpretation—‘that you say how it is’.

Overall, dealing with the patients’ and their relatives’ emotions towards bad news was considered especially challenging. The interviewees stressed the necessity of more practice and teaching of communication skills in medical studies. One possible way would be for students and residents to observe physicians when they deliver bad news to patients, although the conversations are highly sensitive.

As mentioned above, the topic was more prevalent among resident participants. Corresponding differences could also be observed in the depth of the discussion of the topic. While the residents showed a more comprehensive understanding of the topic, the medical students’ descriptions remained at a more general level.

### Caring for Relatives

Seven students and two residents described providing family members with information and answering their questions as physician’s duty. Furthermore, regarding preparation and counselling, interviewees saw their main task in avoiding that relatives are unpleasantly surprised by and can accept the death of their loved one. To ‘have family members on board’ was described as ‘extremely important’ in order to enable family members to experience the death of the loved one as ‘pleasantly’ as possible.

Moreover, our interviewees stressed the importance of offering family members advice on how to design the dying setting and how they could witness the death of their loved one. It was considered important to provide relatives with comprehensive support in various matters—to pass on the address for psychological support and pastoral care, and also help with, for instance, employment concerns.

Our participants, especially residents, deemed caring for family members as challenging and ‘burdensome’ for physicians. The major challenge was seen in the fact that family members are laypersons whose supervision requires resources. Moreover, witnessing the suffering of relatives was described especially burdensome. As one resident stated:

I think personally it’s actually rather stressful. Well, it’s part of the job in a way, but that would be, the dying of the patients would be much easier without having the relatives there. (P.F., resident)

The interviewees also emphasized the importance of the care of relatives for physicians themselves. They agreed that attentive care of relatives makes it easier for physicians to deal with the dying experience.

The topic was more prevalent among the medical students. While the two residents reported mainly on the importance and difficulties of care for relatives, the medical students’ descriptions focused on the elements of good family care.

### Allowing Death

Seven students and six residents addressed as the physician’s duty to allow patients to die. Our interviewees agreed that as a physician, at a certain point one has to make the decision to no longer treat the patient curatively, but rather palliatively.

[I’ve learned] that you also have to let people go, that you don’t always have to do everything until the end. (M.L., resident)

Our interviewees described that seeing no chance for a patient to be cured was the point one should begin to think about allowing death. The identification of this point, however challenging, was primarily connected to the patient’s health status and quality of life. Moreover, consequences for the patient—chances of successful treatment and the risks of a therapeutic option—and the will of the patient were stated as central factors when making decisions.

In this context, various interviewees sharply criticized the maximum therapy approach in hospitals considering the danger of unnecessarily prolonging a patient’s suffering. The interviewees also addressed the danger of missing the moment to talk to the patient about the hopelessness of their situation and, thus, the danger of depriving the patient of the opportunity to die according to their own wishes.

At the same time, the interviewees attempted to explain the excessive use of a maximum therapy approach in hospitals. The participants referred to the prevailing image of the physician as a ‘healer’ and to the physicians’ expectations of themselves as having to cure patients. Furthermore, our interviewees were concerned that reflection on the meaningfulness of therapeutic measures hardly takes place in medical studies. Due to a neglect of the topics of death and the limits of medicine in medical school, alternatives to maximum therapy are insufficiently considered by physicians.

Often, I think, things are done because you can do them and because you don’t learn any other way. You only learn that if some laboratory value is wrong, then you have to do something about it, then you have to look at what’s there and make sure that it’s okay again, and you don’t learn to ask yourself whether it even makes sense. (F.E., student)

The attitude of allowing patients to die goes hand in hand with the shift in attitude towards death from one of rejection to one of acceptance. Various participants described a change from viewing death as ‘the worst thing’ towards regarding it as ‘part of life’. Their experiences had made the interviewees aware that death is ‘something ordinary’ which ‘belongs to life as much as birth’ and will happen to everyone at some point in time. Some interviewees even started to see death as a ‘release’, e.g. from suffering. Several participants described developing an accepting attitude towards death as an actively initiated measure of self-protection.

Overall, our interviewees agreed ‘that healing also includes not being able to heal’ and that one has to become consciously aware of this option. In this context, the interviewees emphasized the importance of reflecting about death as a physicians’ attitude towards it influences their dealing with the dying patients, e.g. an accepting attitude towards death was considered as a prerequisite for allowing patients to die.

### Enabling a Beautiful Death

Seven students and six residents mentioned enabling a *beautiful death* [9] as an essential medical task and discussed different aspects of accompanying patients to a beautiful death. Central elements of this process included ensuring comprehensive medical care and maintaining patients’ quality of life—e.g. by reducing the physical and mental suffering. Also, relieving patients of fears, helping them to find inner peace and mentally preparing patients and their loved ones for the impending death were considered central.

According to our interviewees, accompanying patients to a beautiful death also involves arranging patients’ environment appropriately. This includes that loved ones are present, rituals are carried out and the setting is pleasantly arranged. To create the opportunity for patients to die at home in the vicinity of their family was deemed important. Thus, the aspects described correspond to the characteristics of a *beautiful death*, which were described in detail in the previous study (Jedlicska et al. 2021). Moreover, interviewees considered it important to come to shared decisions with patients and their families, whereby wishes of patients were stated as ‘absolutely crucial’.

Confidence in dealing with dying and death and the absence of fear of death for the physician were discussed as prerequisites for accompanying patients to a beautiful death. Abandoning the attitude of wanting to prevent death at all costs was seen as essential.

### Making Decisions and Taking Responsibility

One medical student and four residents addressed making decisions and taking responsibility for those decisions by backing them with consistent arguments. In this context, a specific image of the physician was described by our interviewees. The physician was compared with God and was pictured as the final authority who ‘has life and death in their hands’. The interviewees found the responsibility for the life and death of the patient to be burdensome.

In addition to the frequency with which the topic is addressed, the differences in the language used by the residents and the medical student are apparent. While residents spoke about the duties of decision-making and accompanying responsibilities in first person, referring to their own roles as physicians, the medical student reported in third person, referring to their physician colleagues at work.

## Discussion

The present study investigated changes in medical students’ and residents’ understanding of their role as (future) physicians in dealing with dying patients and death resulting from encounters with death. Eight themes could be identified in this regard: *thoughtful care*, *distancing*, *keeping in control*, *communication*, *caring for relatives*, *allowing death*, *enabling a beautiful death*, and *making decisions and taking responsibility*.

With regard to thoughtful care, the importance of empathy is recognised as a result of increased exposure to the dying patients [24, 29, 30]. In this context, our results highlight the tension between personal attachment and professional distancing that has already been discussed in the literature [10, 20]. The simultaneous existence of the demands for distancing and emotional involvement with the accompanying fear of loss of control and concern about losing empathy illustrates the uncertainty, especially among medical students. Our findings are consistent with the literature that identifies becoming insensitive as one of the effects of confrontation with death and attributes this development to the profession itself [15]. Moreover, our findings emphasize the great importance of dealing with the question of balance and one’s personal limits of emotional involvement [15].

Furthermore, the importance of emotional and cognitive distancing for self-protection stands out [10]. Our results add another perspective to this finding as they show the importance of distancing for making sober therapeutic decisions and allowing patients to die, and thus for practicing medicine. The impact of personal attachment to patients and their relatives on physicians’ emotional responses to patient’s death has been discussed in previous research [2, 7, 9]. Our study extends these findings addressing the personal attachment as a factor that makes it more difficult to distance oneself from patients’ fates and to maintain control over one’s own emotions.

Rhodes-Kropf et al. (2005) identified *doctors should not have emotional reactions to death* as a central message learned by medical students during their third year rotations [3]. In contradiction to this finding, our interviewees found it perfectly legitimate for physicians to feel emotions themselves. However, they agreed that it is ‘not the task’ of physician to express feelings excessively [31]. The importance of keeping in control in order to ‘stay functional’ was outlined. Corresponding to Boland et al.’s findings, our study emphasizes that keeping oneself under control and managing one’s emotions needs to be learned [24].

Exposure to the dying patients and their relatives made our interviewees recognise the importance of honest, well-timed and complete communication [30, 32, 33]. Honest communication of patients’ health status was seen important to involve patients and their relatives in the decision-making process [29, 33, 34]. Well-timed communication was considered a precondition of affording patients and their loved ones the opportunity to prepare for the impending death [34]. Completeness was regarded crucial in enabling well-informed decision-making. Corresponding to previous research, the challenging nature of delivering bad news was highlighted [5, 15, 35]. A particular challenge when delivering bad news is dealing with the hopes of patients and their relatives [36].

Furthermore, caring for relatives was determined as an essential medical task involving a multitude of aspects [1, 15, 19, 30]. Echoing previous research, our study highlights the challenging character of caring for relatives. In particular, witnessing relatives suffer was perceived as ‘burdensome’ [1, 5, 10, 15]. However, our study highlights the positive effects of relatives’ care on physicians themselves, emphasizing that successfully caring for relatives helps physicians to deal with the experience of patients dying.

As an effect of encounters with death, our study addresses allowing death at a certain point as a physicians’ duty. The decision to let the patient die, especially in finding the right point, was described as particularly challenging. In this context, what stands out is the sharp criticism of a maximum therapy approach. Our study thus addresses the tension between legal regulations and ethical values identified in previous research [15]. The prevailing image of the physician as a ‘healer’, the neglect of death and the lack of discussion of the limits of medicine in medical studies were cited as reasons for the excessive use of such a maximum therapy approach. Corresponding with Rhodes-Kropf et al., the lack of reflection on the meaningfulness of therapeutic measures was criticized [3].

The changes in the view of death as ‘part of life’ as a result of encounters with death has already been described in literature [1, 15, 20, 26, 27, 37, 38]. Our study provides insights into the underlying causes of these changes and identifies the altered view of death as an actively initiated measure of self-protection.

To ensure comprehensive medical care, to mentally prepare patients and their loved ones for the impending death, to arrange the dying environment pleasantly and to enable patients and their loved ones to say goodbye properly were described as central components of accompanying patients to a beautiful death [7, 9, 12, 15, 39]. In this context, experiencing suffering deaths seems to shape students’ and residents’ understanding of their role as physicians, by recognizing the reduction of suffering as an important component of enabling a beautiful death and central medical task. In addition to taking the patient’s wishes into account, the literature also emphasizes the importance of the patient’s quality of life and the concept of dying with dignity [15].

Moreover, making therapeutic decisions confidently and taking responsibility for those decisions were described as a physician’s duty. Our findings are consistent with Aase et al.’s findings, which showed that physicians found being responsible for decisions and treatments both rewarding and burdensome [40].

In comparison, distinctly more residents than students commented on the topics of communication and making decisions and taking responsibility. The topics of thoughtful care and caring for relatives were of greater concern among medical students. Compared to the students, who predominantly focused on describing the elements of good care, the residents drew attention to the relevance and burdensome nature of this task. The lack of decision-making power and lower level of responsibility associated with the hierarchical status of medical students may be the cause of students’ focus on the more psychosocial components of care. The validity of these hypotheses should be investigated in future studies.

In summary, our findings show that encounters with dying and death critically shape medical trainees’ understanding of their role as a (future) physician in dealing with dying patients and death. Our study highlights the complex nature of this development and identifies the challenges medical students and residents face in forming their professional identity in relation to dealing with dying and death. In this way, the study highlights the entry points for actively supporting (future) physicians in this process and in negotiating the question of what makes a good physician in dealing with dying patients and death.

### Implications for Practice

Alongside the criticism of an overly curative orientation of medical studies, the call to give the topic of death more prominence stands out in our findings [15, 21, 41, 42]. In addition to the themes already raised in the discussion, we here present some suggestions on increasing awareness of the topic.

Our study emphasizes the importance of allowing patients to die and finding the right point in time to do so as very challenging. We encourage active engagement with the subject of allowing death and with the existential question of when is the right time to accept that death is inevitable and that patients should be allowed to die, starting in medical school.

Echoing Jackson et al.’s findings [7], our study highlights the distressing nature of witnessing the excessive use of a maximum therapy approach in hospitals. Thus, our results call for an active discussion of personal limits and the limits of medicine in the context of medical school as well as for more intensive reflection on the meaningfulness of therapeutic measures.

Our interviewees agreed on communication being ‘essential’ when dealing with patients and criticized that teaching communication skills was underestimated in medical studies [30, 34, 43]. Echoing students’ voices in previous research [24, 44], our interviewees emphasized the importance of *experiential learning* and suggested accompanying and observing senior and experienced physicians when delivering bad news to patients. The greater confidence in one’s own communication skills as an effect of accompanying physicians while interacting with relatives has already been described in the literature [10]. Another recommendation would be to facilitate real-world patient interactions, which has been shown to have a positive impact on students’ communication skills and attitudes, e.g. students’ ability to face severely ill patients without fear [44].

The described picture of physicians as a final authority responsible for the patient’s life and death illustrates the burdensome nature of physicians’ responsibility in making therapeutic decisions and taking responsibility for them. Given the burdensome nature of this responsibility, it would be advisable to pay special attention to these issues during medical school.

### Limitations of This Study and Suggestions for Further Research

Several limitations of our study should be mentioned. Due to the qualitative methodological approach, extrapolating the findings of this study to other contexts may be difficult. However, we aimed to ensure the *intersubjective traceability of the research process* which is considered central to being able to capture the quality of a study regarding the decoding of the evidence [45], as well as to enhance the *transferability*, which refers to the applicability of the qualitative research findings to other social contexts and settings [46]. For this purpose, we documented the individual steps of the research process—sampling, data collection and data analysis—in detail. Moreover, the data interpretation in small groups of researchers ensured the quality of the research.

Another considerable limitation is the fact that the data were collected about 9 years ago. Although the medical curriculum has not changed significantly since then, it can be assumed that encounters with death in the context of the COVID-19 pandemic have had an impact on medical students’ and residents’ attitudes and understanding of their roles as (future) physicians. This impact should be investigated in future studies. Subsequently, the current study can serve as a comparative foil to uncover the effects of the COVID-19 pandemic on physicians’ attitudes towards dying and death.

Although we believe that theoretical saturation has been reached in relation to certain themes, e.g. communication, and therefore no further data collection is necessary, we believe that additional data collection on other themes, e.g. making decisions and taking responsibility, possibly with a larger sample size, could provide new insights.

There is evidence that various factors, including cultural and religious background, influence medical students’ attitudes towards death [42, 47]. Our study has shown that attitudes towards death impact (future) physician’s dealing with the dying patients. Therefore, cultural and religious background can be expected to influence the impact of encounters with death on the understanding of the role of physician in dealing with dying patients. This impact should be investigated in detail in further studies.

## Conclusions

Our study highlights the formative impact of encounters with death on medical students’ and residents’ perception and shaping of their role as (future) physicians in dealing with dying patients and death. The change in the view of death as ‘part of life’, and in attitudes towards an acceptance of death was identified. Moreover, the shift in understanding the physicians’ role from cure- to care-oriented was revealed. In this context, the role expectations that physicians encounter when dealing with dying patients were defined.

## Data Availability

The datasets generated during and/or analysed during the current study are not publicly available due to privacy and confidentiality but are available from the corresponding author on reasonable request.

## Appendix

**Interview guideline used in interviews with final-year medical students and midterm residents**

*Opening question*

• Can you recall an experience that involved dying and death, which influenced you the most?

*Follow-up question*

• Can you describe in detail this experience/relationship/person?

***Supplementary questions***

For further elaboration of different dimensions:

• What were your thoughts during this event/experience/relationship, and so forth?

• What kinds of emotion did this event/experience/relationship, and so forth evoke in you?

• What did you learn from this experience?

To capture the consequences of the described experiences regarding different dimensions:

• How did this experience affect your personal attitude toward dying and death?

• How did this experience affect your understanding of your role as a (future) physician in dealing with dying patients and death?

• How did this experience affect your behaviour toward dying patients and their relatives?
